# Estimating personal exposures to household air pollution and plastic garbage burning among adolescent girls in Jalapa, Guatemala

**DOI:** 10.1016/j.chemosphere.2023.140705

**Published:** 2024-01

**Authors:** Katherine A. Kearns, Luke P. Naeher, John P. McCracken, Dana Boyd Barr, Eri Saikawa, Mayari Hengstermann, Erick Mollinedo, Parinya Panuwet, Volha Yakimavets, Grace E. Lee, Lisa M. Thompson

**Affiliations:** a University of Georgia, Department of Environmental Health Science, College of Public Health, Athens, GA, USA; b Center for Health Studies, Universidad del Valle de Guatemala, Guatemala City, Guatemala; c Gangarosa Department of Environmental Health, Rollins School of Public Health, Emory University, Atlanta, GA, USA; d Nell Hodgson Woodruff School of Nursing, Emory University, Atlanta, GA, USA

**Keywords:** Exposure assessment, Household air pollution, Endocrine disrupting compounds, Biomonitoring, Plastic pollution

## Abstract

Waste collection services are uncommon in rural areas of low-resource countries, causing waste accumulation and subsequent dumping and burning of garbage. Air pollution from household garbage burning, including plastics, has been observed in Jalapa, Guatemala in addition to household air pollution (HAP) from cooking. Adolescent girls often help with these cooking and household tasks, but little is known about their exposures. We characterized 24-h exposures to HAP and household garbage burning in adolescent girls by measuring fine particulate matter (PM_2.5_), black carbon (BC), urinary biomarkers of polycyclic aromatic hydrocarbons (PAHs), bisphenol A (BPA), and phthalates. We recruited 60 girls between 13 and 17 years of age who helped with cooking activities and lived with participants of the Household Air Pollution Intervention Network (HAPIN) trial. We recruited n = 30 girls each from the control (wood-burning stove) and intervention (liquefied petroleum gas stove) arms. We also measured real-time kitchen concentrations of BC in 20 homes (33%). PM_2.5_ and BC were measured in n = 21 control and n = 20 intervention participants. Median concentrations of personal PM_2.5_ and BC and kitchen BC were lower (p < 0.05) in the intervention arm by 87%, 80%, and 85%, respectively. PAH metabolite concentrations were lower (p < 0.001) for all nine metabolites in intervention (n = 26) compared to control participants (n = 29). Urinary BPA concentrations were 66% higher in participants who reported using cosmetics (p = 0.02), and phthalate concentrations were 63% higher in participants who had reported using hair products during the sample period (p = 0.05). Our results suggest that gas stoves can reduce HAP exposures among adolescents who are not primary cooks at home. Biomarkers of plastic exposure were not associated with intervention status, but some were elevated compared to age- and sex-matched participants of the National Health and Nutrition Examination Survey (NHANES).

## Highlights

• Household garbage burning common waste disposal method in Jalapa, Guatemala.
• Plastic waste presents opportunity for chemical exposures in adolescent girls.
• Urinary biomarkers elevated in participants in Jalapa compared to U.S. survey.
• Few studies report pollutant exposures in adolescents in low-resource settings.

## Graphical abstract

**Figure:**
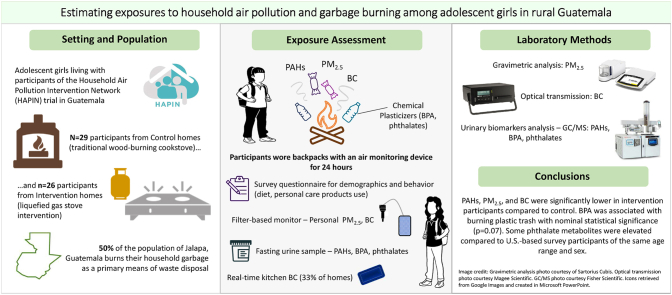

## 1 Introduction

Household air pollution (HAP) is generated from the use of solid or “biomass” fuels (coal, charcoal, wood, animal dung, and agricultural crop residues) in open fires or inefficient cookstoves in or near the home (Johnson et al., 2021; Zhang and Smith, 2007). The majority of the approximately 3.8 billion people impacted by HAP live in low- and middle-income countries (LMICs) (Health Effects Institute, 2020). The smoke that results from the incomplete combustion of biomass fuels is a heterogeneous mixture of harmful air pollutants including fine particulate matter (PM_2.5_), black carbon (BC), and polycyclic aromatic hydrocarbons (PAHs)(Arif et al., 2018; Zhang et al., 2019).

Another form of HAP exposure besides cooking activities is household garbage burning. This is of particular concern in rural areas of LMICs, where there is a general lack of infrastructure for waste collection facilities (Edjabou et al., 2012; Hiramatsu et al., 2009; Lemieux et al., 2004; Ramadan et al., 2022; Wiedinmyer et al., 2014; Zeng et al., 2016). Waste collection rates in rural LMICs are estimated at 33% (Kaza et al., 2018), leaving approximately two billion people worldwide without access to formal waste disposal options (Velis and Cook, 2021). Waste generation is expected to more than double globally by 2050 if the current trajectory is maintained (Kaza et al., 2018), which will certainly strain areas already struggling with waste accumulation.

In Guatemala, HAP from both cooking activities and household garbage burning are prevalent. Approximately 50% of the total population and 86% of the rural population, including the city of Jalapa, primarily relies on “polluting fuel” sources including biomass for cooking and heating [dataset] (World Health Organization, 2023). According to the Guatemala Population and Housing Census of 2018, 50% of the population of Jalapa burns their household waste on or near their property as a primary means of waste disposal [dataset] (National Institute of Statistics Guatemala, United Nations Population Fund UNFPA, 2018), likely due to the lack of infrastructure for waste management services. According to Hettiarachchi et al. (2018), only 25% of the total population of Guatemala has access to solid waste disposal services such as landfills and controlled dumps. Although open burning of household garbage presents a convenient way to self-manage waste, it increases the risk of harmful pollutant exposures through water, soil, and air (Ferronato and Torretta, 2019; Ramadan et al., 2022; Velis and Cook, 2021).

A recent systematic review conducted by Velis and Cook explored the potential impacts of residential garbage burning on human health and synthesized evidence into eight groups of substance emissions, including PM_2.5_, PAHs, BPA, and phthalates. They found that individuals who were routinely in close proximity to burn piles, especially informal waste reclaimers (also referred to as “waste pickers”), were at the highest risk of exposure to an array of health-damaging air pollutants (Velis and Cook, 2021). This has implications for families who burn their own trash near their homes, though exposure assessment studies were not included in the review.

PM_2.5_, BC, and PAHs are products of incomplete combustion generated from open waste burning and are associated with an array of adverse health effects (Arku et al., 2020; Dutta et al., 2020; Woolley et al., 2020; Velis and Cook, 2021). The chemical composition of the smoke from household garbage burning depends on a variety of factors including type of waste burned, environmental conditions, and burn frequency (Lemieux et al., 2004). A recent publication by Bardales Cruz and colleagues characterized emissions from domestic burning of plastic waste across 22 departments in Guatemala. They found that as much as 30% of organic carbon, 24% of BC, 24% of PM_2.5_, and 2% of CO_2_ emissions in Guatemala might not be accounted for by excluding open plastic burning from emissions inventories. Furthermore, the burning of plastic waste releases an array of other pollutants of potential health concern including ammonium, chloride, nitrate, sulfate, antimony, and other trace elements (Bardales Cruz et al., 2023).

Other pollutants generated by household garbage burning are plasticizers and chemical additives such as phthalates and BPA. These chemicals are commonly added to plastic products to improve texture and rigidity and have the potential to leach into the environment as a result of incomplete combustion (Velis and Cook, 2021). Phthalates are also added to many consumer products including personal care products like soaps and hair products (Benjamin et al., 2017), pesticides, toys, and cosmetics (Zhang et al., 2022b). Long-term exposure to phthalates has been shown to impact pregnancy (Grindler et al., 2018; Wang and Qian, 2021), child growth and development, and reproductive systems in young children and adolescents (Benjamin et al., 2017; Wang and Qian, 2021). Exposure to these chemicals can occur through ingestion, inhalation, or dermal contact (Benjamin et al., 2017).

BPA is a plasticizer added to products such as baby bottles, water bottles, food and beverage containers, toys, glasses, and CDs (Ma et al., 2019). Exposure can occur dermally or through the ingestion of food or drinks that have been packaged in or come into contact with BPA (Cimmino et al., 2020; Geens et al., 2009; Grindler et al., 2018). BPA interacts with various biological receptors including estrogen receptors and has been associated with breast cancer development (Ma et al., 2019). Both BPA and phthalates have been proposed in the etiology of polycystic ovarian syndrome (PCOS) in adolescent girls (Akgül et al., 2019). Inhalation exposures are also possible through exposure to BPA-containing dust particles or through burning products made with BPA, though more studies are needed to understand the associated health impacts upon exposure (Abraham and Chakraborty, 2020; Vasiljevic and Harner, 2021).

HAP exposure assessment and health effects studies related to cooking practices in Guatemala are abundant in the literature (Johnson et al., 2021; McCracken John P. et al., 2007; Pope et al., 2015; Smith et al., 2010, 2011; Weinstein et al., 2017, 2020). Women and children are the populations typically prioritized in HAP studies because they typically spend more time at home and near the stove, compared to men (Hollada et al., 2017). However, limited evidence exists in the literature of HAP exposure assessment in young adults or adolescents (Huang et al., 2019; Murawski et al., 2020; Yang et al., 2022; Zhang et al., 2022a), despite the knowledge that adolescent girls often help with household tasks including cooking and garbage burning. HAP exposure assessment studies in adolescent girls in LMICs, where residential biomass and garbage burning are both prevalent, are even less common (Huang et al., 2019).

Given the ubiquity of plastic waste and the prevalence of HAP and household garbage burning in Guatemala, we sought to characterize exposures experienced by adolescent girls in Jalapa, Guatemala to PM_2.5_, BC, PAHs, phthalates, and BPA. For this pilot study, we leveraged the Household Air Pollution Intervention Network (HAPIN) randomized controlled trial to recruit girls from the control (biomass stove) and intervention (liquefied petroleum gas; LPG stove) arms to compare exposures. We assessed their exposures to PM_2.5_ and BC, collected real-time kitchen area BC concentrations, and analyzed urinary biomarkers of exposure to PAHs (markers for both HAP and garbage burning), and biomarkers of phthalates and BPA to assess exposures to plastics. We also collected survey data to assess other potential dietary, dermal, and inhalation exposures to measured pollutants. This pilot study aims to address important knowledge gaps in personal exposures to HAP and garbage burning in adolescent girls in rural areas of LMICs.

## 2 Methods

### 2.1 Study setting

This pilot study was nested within the HAPIN trial, which has been described in detail previously (Clasen et al., 2020, 2022; Johnson et al., 2021) and summarized briefly here. The HAPIN trial took place between 2017 and 2022 in four LMICs: Guatemala, India, Peru, and Rwanda. HAPIN households (n = 3200 total, n = 800 per study site) were randomized into either the control arm, continued use of a biomass stove (n = 1607), or the intervention arm, free LPG stove and gas for 18 months (n = 1593). The HAPIN trial characterized 24 h personal exposures in pregnant women and kitchen concentrations of PM_2.5_ and BC pre- and post-intervention. For this pilot study, we recruited 60 adolescent girls residing in HAPIN households at the Guatemala site in rural Jalapa.

The protocol for this study has been reviewed and approved by institutional review boards (IRBs) or Ethics Committees at Emory University (00109014) and Universidad del Valle de Guatemala (198-05-2019). The HAPIN trial has been registered with ClinicalTrials.gov (Identifier NCT02944682). All participants provided written informed consent.

### 2.2 Inclusion and exclusion criteria

We sought to recruit 60 girls, 30 each from the control and intervention arms. Researchers were blinded to intervention status through the data collection period and until a certain stage of data analysis. Inclusion criteria were female, ages 13–17 years, residence in households participating in the HAPIN trial, and routine participation in cooking meals at home. Exclusion criteria were known pregnancy, self-reported current cigarette smoker, or plans to move temporarily or permanently outside the study area during the time of the study.

### 2.3 Questionnaire

In total, 56 of the 60 participants met eligibility criteria for the study. Two of the four ineligible participants did not meet age criteria and two did not regularly participate in cooking activities. An additional participant refused to provide a urine sample, and her instrument also failed during the air monitoring period, leaving us with a total sample size of 55 participants. Written consent to participate in the study was provided by a parent or guardian at the time of recruitment. All eligible participants were informed of study objectives, time commitment, benefits, and risks of the study.

Local, trained fieldworkers verbally administered a baseline survey questionnaire in Spanish to collect sociodemographic information, household and stove characteristics, perception of waste generation and disposal in the community, health effects related to HAP exposure, and sources of HAP in and outside the home. Another survey was administered the following day when urine samples were collected to determine potential sources of exposure that occurred during the previous 48 h (24 h during the monitoring period and the 24 h preceding the monitoring period), to best attempt to capture the various exposure types). Responses were recorded on tablets and saved on REDCap™ (Harris et al., 2009, 2019).

### 2.4 Exposure assessment and sampling strategy

#### 2.4.1 Personal exposure assessment and laboratory analysis of PM_2.5_

Personal exposure to PM_2.5_ was assessed on all participants using the Triplex Personal Sampling Cyclone (Mesa Labs) and Casella Tuff Pro pump (Casella, Buffalo, NY, USA). Pre-weighed Teflon filters with a diameter of 37 mm (Pall Corporation, Port Washington, NY, USA) were loaded into a plastic cassette (SKC Limited, United Kingdom) and affixed to the aluminum cyclone. The cyclone and filter apparatus were connected to the pump via tubing. The pump/cyclone system weighs about 450 g total and was calibrated at a flow rate of 1.5 L min^−1^. The instruments were calibrated before each deployment, and the flow rate was recorded at the field station before and after the sampling period to determine the average sample flow rate.

At the start of the sample period in the home, trained field staff turned on the air sampling equipment and placed the pump into a small backpack. The tube and cyclone were affixed along the strap of the backpack such that the filter was placed in the participant's approximate breathing zone. The participant was asked to wear the backpack at all times, unless she were to engage in an activity which might damage the equipment such as bathing or sleeping and was instructed to keep the backpack within a meter of her person during these activities.

The instruments were stopped by a field technician when they returned after 24 h to collect the equipment. The sampling equipment was carried back to the field office and filters were removed from the cyclones in a clean laboratory. Filters were placed in labeled petri dishes and refrigerated at 4 °C until they were hand-carried in coolers with blue ice by a traveler to the University of Georgia (UGA; Athens, GA, USA) for analysis. We followed the HAPIN protocol for gravimetric analysis of PM_2.5_ and optical transmission of BC for personal exposure assessment, as described in detail elsewhere (Johnson et al., 2020).

#### 2.4.2 Measurement of real-time kitchen area BC

We measured 24-h real-time BC concentrations in the kitchens of 20 participants (n = 10 control and n = 10 intervention homes) using the Model AE51 microAeth Black Carbon aerosol monitor (Aethlabs, Inc., range: 0–1 mg m^−3^, resolution: 0.001 mg m^−3^, flow rate: 50 mL min^−1^). The microAeth was deployed in the kitchen 0.5 m above the ground near the stove and was loaded with a Teflon-coated glass fiber filter strip prior to deployment (Pallflex Fiberfilm T60A20, Pall Life Sciences, MI, USA). The monitor draws air into an inlet 3 mm in diameter with a built-in pump and measures the rate of light absorption as aerosols deposit on the filter at a flow rate of 50 mL min^−1^ and a time interval of 60 s. The absorbance of the sample area of the filter is therefore measured once every 60 s relative to a reference portion of the filter paper. These parameters were chosen based on the manufacturer's instructions to optimize performance of the microAeth in this particular study setting. The BC data was post-processed using an Optimized Noise-reduction Averaging (ONA) algorithm as recommended by the manufacturer.

#### 2.4.3 Urinary biomarkers of PAHs, phthalates, and BPA

The phthalates of interest for this study are commonly described in the literature (Benjamin et al., 2017; Radke et al., 2020) and include metabolites of the parent compounds di(2-ethylhexyl) phthalate (DEHP), dibutyl phthalate (DBP), butyl benzyl phthalate (BBP), and diethyl phthalate (DEP). Thus, the measured metabolites for this study were mono-*n*-butyl phthalate (MBP), monobenzyl phthalate (MBzP), mono-2-carboxymethylhexyl phthalate (MCMHP), mono-2-ethyl-5-carboxypentyl phthalate (MECPP), mono-2-ethyl-5-hydroxyhexyl phthalate (MEHHP), mono-2-ethylhexyl phthalate (MEHP), mono-2-ethyl-5-oxohexyl phthalate (MEOHP), mono-ethylhexyl phthalate (MEP), mono-isobutyl phthalate (MiBP).

The metabolites measured in this study include 1-napthol, 2-naphthol, 2- and 3-hydroxyfluorene, 1-hydroxypyrene, and 1-, 2-, 3-, and 4-hydroxyphenanthrene. PAHs like these that are composed four rings or less (classified as low molecular weight PAHs) are more readily excreted in the urine (Yang et al., 2021), and thus were the target metabolites for this study.

Participants were instructed to collect the first urine in the morning at the end of the 24-h sample period in a provided plastic urine collection container (previously tested to be BPA and phthalate-free). Field blanks were collected alongside of urine samples for quality assurance purposes to assess potential field contamination. Urine samples were collected from all participants when the technicians returned to pick up air monitoring equipment. Samples were transported to the field laboratory and were processed within 8 h of collection. Samples were stored in the laboratory's freezer at −20 °C until shipment to Emory University within 6 months of collection (Atlanta, GA, USA).

All urine samples were randomized using a Fisher-Yates shuffling algorithm prior to analysis to reduce any potential batch effects. A 0.5-mL aliquot of urine was spiked with isotopically labeled analogues of the target phthalates and phenols and then was subjected to an enzyme hydrolysis to liberate glucuronide-bound conjugates. The hydrolysate was extracted using an ABS Elut-NEXUS solid phase extraction column, eluting with acetonitrile and ethyl acetate. The extract was concentrated to dryness and reconstituted in mobile phase for analysis using liquid chromatography-tandem mass spectrometry (LC-MS/MS) using two separate injections and acquisition methods.

Analyte concentrations were calculated using isotope dilution calibration. Two quality control materials (one high and one low) and one blank sample were analyzed concurrently with each set of 28 unknown samples. Further quality assurance measures were included in the sample analyses including the analysis of NIST SRM 3672 and 3673 (one of each per 50 samples), and bi-annual participation in the German External Quality Assessment Scheme (G-EQUAS). Specific gravity was measured using a refractometer.

All metabolite concentrations were adjusted for measured creatinine concentrations to account for variability in the volume of urine and the concentrations of endogenous and exogenous chemicals from void to void (Barr et al., 2005).

### 2.5 Statistical analysis

All statistical analyses were performed in R version 4.0 and later (R Foundation for Statistical Computing, 253 Vienna, Austria). We calculated descriptive statistics for each of the pollutants. For the urinary biomarkers (phthalates, BPA, and PAHs; n = 55) we calculated the geometric mean (95% confidence interval; CI), median (interquartile range; IQR), the 90th and 95th percentiles, and the minimum and maximum concentrations. For the air monitoring data (personal PM_2.5_ and BC and kitchen BC), we calculated the median and IQR by study arm. For PM_2.5_, we also evaluated the percentage of samples that were below the World Health Organization's Annual Interim Target 1 (IT-1) for exposures, which is 35 μg/m^3^ (World Health Organization, 2021).

Differences in exposures between assigned treatment arm were evaluated using non-parametric tests (Wilcoxon Rank Sum), given that the data did not follow the assumptions of normality. Biomarker data were natural log-transformed before testing associations between reported dermal, ingestion and inhalation exposures and measured pollutant concentrations using linear regression.

For the biomarker data, we also compared metabolite concentrations of PAHs, phthalates, and BPA between our participants (stratified by study arm) and age- and sex-matched participants of the National Health and Nutrition Examination Survey (NHANES), a U.S.-based survey.

## 3 Results

### 3.1 Household and participant characteristics

Household and participant characteristics, as well as exposures to HAP and plastics stratified by study arm, are provided in Table 1. Participants were split 53% control and 47% intervention. The median age for each group was similar (14–15 years), and the majority of participants (65%) were currently in school. Most participants (98%) reported feeling that their community has a waste disposal issue and that there was nowhere to properly dispose of accumulated garbage.

**Table 1:** Participant demographics (n = 55) and reported dermal, ingestion, and inhalation exposures to air pollution and personal care product use over previous 48 h stratified by study arm.

	Control (Biomass Stove) N (%) or Median (IQR)	Intervention (LPG Stove) N (%) or Median (IQR)
***Participant characteristics***		
Number of participants	29 (53)	26 (47)
Age in years	15 (14–16)	14 (14–15)
Currently in school	18 (62)	18 (69)
Household uses LPG exclusively	N/A	20 (77)
Household burns plastic in wood fire at least 2x/week	14 (48)	6 (23)
Burning trash is home's preferred waste disposal method	23 (79)	19 (73)
***Product use over past 48 h***		
**Dermal**		
Cosmetics†	9 (31)	6 (23)
Perfume	11 (38)	11 (42)
Lotion	20 (69)	14 (54)
Hair products††	17 (59)	13 (50)
Sunscreen	0 (0)	1 (4)
Deodorant	16 (55)	13 (50)
Baby powder	1 (3)	0 (0)
**Ingestion**		
Ate grilled food	4 (14)	3 (12)
Ate charred/burnt tortillas		
0	13 (24)	13 (50)
1–4	10 (34)	9 (35)
5+	6 (21)	4 (15)
Used plastic plates for eating and/or reheating food	17 (59)	15 (58)
Used plastic cups	17 (59)	16 (62)
Used plastic utensils for eating	4 (14)	4 (15)
Drank water packaged in a plastic bag	5 (17)	2 (8)
Drank canned beverage	2 (7)	1 (4)
Drank beverage packaged in a plastic bottle	7 (24)	4 (15)
**Inhalation**		
Was around an open fire	28 (97)	9 (35)
Plastic was burned in the open fire	5 (17)	2 (8)
Plastic was used to ignite the fire (accelerant)	5 (17)	2 (8)
Burned trash in outdoor burn pile	12 (41)	1 (4)
Trash burned included plastic trash	10 (34)	0 (0)

*N:* number of samples; IQR: interquartile range; LPG: liquefied petroleum gas.

† Cosmetics include foundation, compact powder, blush, eyeliner, mascara, lipstick, eyeshadow, and nail polish.

†† Hair products include gel, straightening products, conditioner, and/or shampoo.

Burning trash was reported to be the preferred method of household garbage management by 76% of participants, which was similar between study arm (79% of control homes and 73% of intervention homes). Participants observed the most trash on the main road in their respective communities (82%), compared to their own yards, church, school, and public land. The most commonly observed types of trash reported by participants were snack wrappers (47%) and plastic bottles (33%).

### 3.2 Participant reported exposures over previous 48 h

After the 24-h sample period, the field staff returned to collect exposure monitors and urine samples and administer questionnaires. At this visit, participants were asked to recount their exposures over the previous 48 h (during the 24-h sample period as well as the day before sampling began). These results are presented in Table 1.

Nearly all control participants (n = 28; 97%) and nine of the intervention participants (35%) reported that they had been around an open fire within the previous 48 h. This could have been interpreted as the cooking fire (particularly for control participants), or any other open fire inside or outside the home. The average time spent around the open fire was 63 min (range: 1–420 min) for control participants and 8 min (range: 0–60 min) for intervention participants (results not shown). Twelve control (41%) and 1 intervention participant (4%) reported they had burned garbage in an outdoor burn pile, and 10 of those 12 control participants reported that the trash included plastic. The average amount of time spent burning trash among the control participants was 9 min (range: 1–30 min), and the single intervention participant reported spending 10 min burning trash (results not shown).

### 3.3 Urinary biomarkers analysis

For the biomarkers analysis, one sample was missing due to participant refusal to provide the urine sample, resulting in a total sample size of 55. Descriptive statistics for BPA, phthalates, and PAH urinary metabolite concentrations are provided in Table 3.

#### 3.3.1 PAHs

**Fig. 1:**
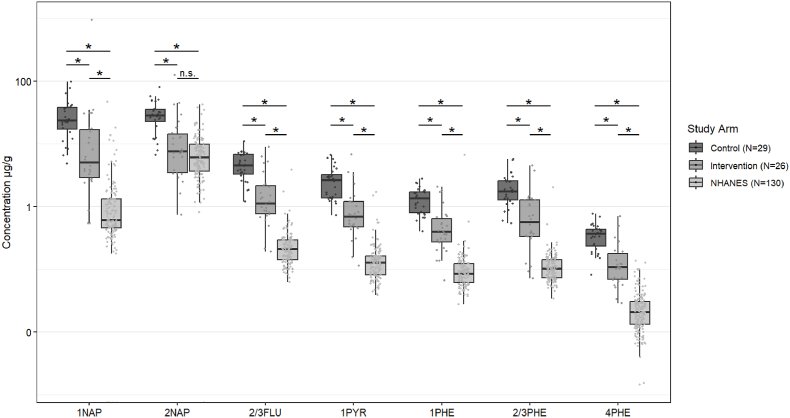
Creatinine-adjusted urinary PAH metabolite concentrations in n = 55 adolescent girls compared to age- and sex-matched participants from NHANES 2015–2016 (n = 130). Statistical significance is indicated (*) at p < 0.05 and non-significance is denoted as “n.s.” 1NAP: 1-naphthol; 2NAP: 2-naphthol; 2/3FLU: 2- and 3-hydroxyfluorene; 1PYR: 1-hydroxpyrene; 1PHE: 1-hydroxyphenanthrene; 2/3PHE: 2- and 3-hydroxyphenanthrene; 4PHE: 4-hydroxyphenanthrene.

Nine hydroxylated urinary PAH metabolites were analyzed, and results are shown in Fig. 1. Due to the inability to isometrically separate some of the metabolites, 2- and 3-hydroxyfluroene and 2- and 3-hydroxyphenanthrene were combined to equal seven total individual measurements. PAH metabolite concentrations were lower (p < 0.001) for each of the seven metabolites in intervention compared to control participants. Conversely, metabolite concentrations were significantly higher in our participants regardless of study arm compared to age- and sex-matched participants of NHANES (n = 130), with the exception of 2-naphthol, where only the control arm was significantly higher than NHANES.

#### 3.3.2 Phthalates and BPA

**Fig. 2:**
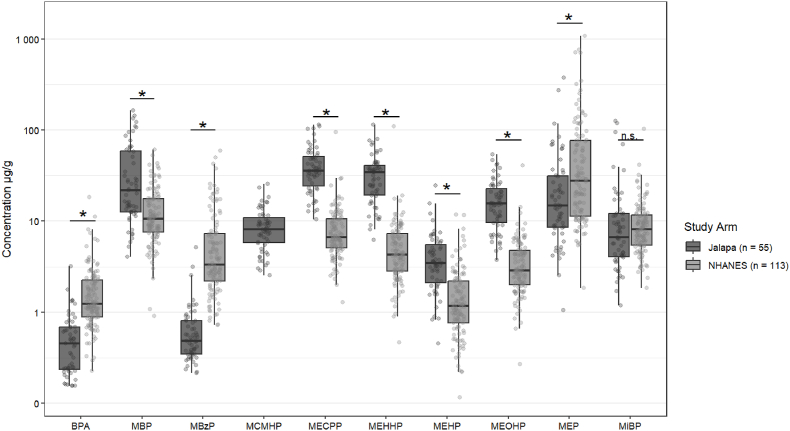
Creatinine-adjusted urinary BPA and phthalate metabolite concentrations in adolescent girls (n = 55) compared to age- and sex-matched participants from NHANES 2017–2018 (n = 113). Statistical significance is indicated (*) at p < 0.05 and non-significance is denoted as “n.s.” BPA: bisphenol A; MBP: mono-*n*-butyl phthalate; MBzP: monobenzyl phthalate; MCMHP: mono-2-carboxymethylhexyl phthalate; MECPP: mono-2-ethyl-5-carboxypentyl phthalate; MEHHP: mono-2-ethyl-5-hydroxyhexyl phthalate; MEHP: mono-2-ethylhexyl phthalate; MEOHP: mono-2-ethyl-5-oxohexyl phthalate; MEP: mono-ethylhexyl phthalate; MiBP: mono-isobutyl phthalate.

Phthalate and BPA exposures were not statistically different between study arms, thus results were pooled and compared to NHANES participants (Fig. 2). Metabolite concentrations were elevated (p < 0.001) for MBP, MECPP, MEHHP, MEHP, and MEOHP in our participants compared to NHANES. We did not observe significant differences between our participants and NHANES participants for MiBP. For MEP, MBzP, and BPA, NHANES levels were significantly higher (p < 0.05) compared to our participants.

### 3.4 Air monitoring data for PM_2.5_ and BC

We analyzed personal PM_2.5_ and BC exposures from 41 participants (n = 21 control, n = 20 intervention) and real-time BC in a subset of 20 kitchens.

#### 3.4.1 Personal exposures to PM_2.5_ and BC

**Fig. 3:**
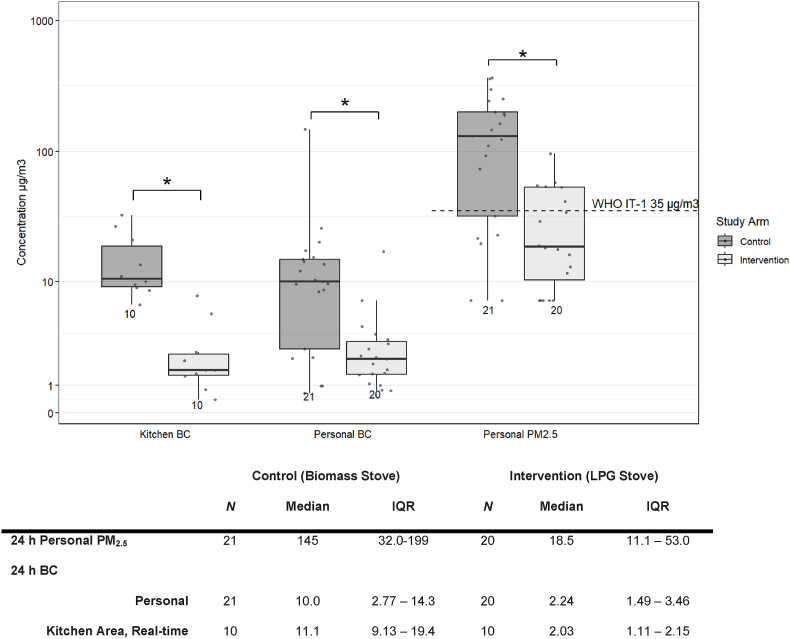
Boxplot and table of 24-h real-time kitchen area BC and time-integrated personal BC and PM_2.5_ stratified by study arm. Sample sizes are given below each box and whisker. Statistical significance (*) is denoted at p < 0.05. The dashed line represents the World Health Organization annual interim-target 1 (IT-1) for PM_2.5_ (World Health Organization, 2021). LPG: liquefied petroleum gas.

We analyzed personal PM_2.5_ and BC exposures from 41 participants (n = 21 control, n = 20 intervention; Fig. 3). Fifteen filters (27%) were removed from analysis due to instrument failure and thus, inability to determine total sample duration and final concentration. There was a significant reduction (Wilcoxon Rank Sum, p < 0.001) in personal PM_2.5_ exposures in intervention participants (median 18.5 μg/m^3^, IQR 11.1–53.0) compared to control participants (median 145 μg/m^3^, IQR 32.0–199). Similarly, there was a significant reduction (Wilcoxon Rank Sum, p < 0.05) in personal BC exposures in intervention (median 2.24 μg/m^3^, IQR 1.49–3.46) compared to control participants (median 10.0 μg/m^3^, IQR 2.77–14.3). Compared to the WHO Annual IT-1 of 35 μg/m^3^ for PM_2.5_, 65% of intervention participants and 29% of control participants and were below the guideline concentrations.

#### 3.4.2 Kitchen area BC concentrations

Due to instrument availability, real-time BC was measured in 20 kitchens (36%). Kitchen area BC concentrations were about five times lower on average in intervention (n = 10) compared to control kitchens (n = 10) (Fig. 3). Median concentrations were similar for personal and kitchen BC concentrations in the control participants (10.0 vs. 11.1 μg/m^3^) and the intervention participants (2.24 vs. 2.03 μg/m^3^), despite using different instrumentation and sampling methods.

### 3.5 Modeling results

Table 2 summarizes the regression results of associations between different dermal, ingestion, and inhalation exposures and measured pollutant concentrations. Pollutant concentrations were natural log (ln)-transformed given the non-normal distribution of the data. For linear regression, ln-transformed pollutants were the dependent (y) variable, and the independent (x) variables were participant-reported exposures from the survey questionnaire.

**Table 2:** Associations between reported dermal, ingestion, and inhalation exposures with air pollutant and chemical plasticizer concentrations over 48 h period. *Note:* We did not test for associations if fewer than five participants reported affirmative exposures. This included use of sunscreen, insecticide, baby powder, or drinking at least one canned beverage during the 48-hr timeframe. CI: 95% Confidence Intervals. % diff: Percent Difference.

		Air Sampler Data				Urinary Biomarker Data					
		Personal PM_2.5_		Personal BC		ΣPAH Metabolites		BPA		ΣPhthalate Metabolites	
		% diff	95% CI	% diff	95% CI	% diff	95% CI	% diff	95% CI	% diff	95% CI
***Dermal***	“Yes” (% total)										
Cosmetics^1^	15 (28)	*Not Tested*		*Not Tested*		*Not Tested*		**66**†	(10, 152)	8	(−37, 86)
Perfume	22 (41)	*Not Tested*		*Not Tested*		*Not Tested*		10	(−26, 64)	33	(−19, 116)
Lotion	34 (63)	*Not Tested*		*Not Tested*		*Not Tested*		11	(−26, 66)	8	(−34, 79)
Hair products^2^	30 (56)	*Not Tested*		*Not Tested*		*Not Tested*		<1	(−36, 40)	**63**†	(2, 161)
Deodorant	29 (54)	*Not Tested*		*Not Tested*		*Not Tested*		<1	(−33, 47)	8	(−34, 75)
***Ingestion***											
Grilled food	7 (13)	*Not Tested*		*Not Tested*		−5	(−61, 127)	*Not Tested*		*Not Tested*	
Burnt Tortillas	29 (54)	*Not Tested*		*Not Tested*		−16	(−54, 52)	*Not Tested*		*Not Tested*	
Plastic plates	32 (59)	*Not Tested*		*Not Tested*		*Not Tested*		21	(−19, 80)	<1	(−39, 64)
Plastic cups	32 (59)	*Not Tested*		*Not Tested*		*Not Tested*		4	(−30, 55)	−17	(−50, 36)
Plastic utensils	8 (15)	*Not Tested*		*Not Tested*		*Not Tested*		3	(−40, 79)	−22	(−60, 55)
Bagged water	7 (13)	*Not Tested*		*Not Tested*		*Not Tested*		−22	(−65, 74)	14	(−60, 228)
Plastic bottle drink	11 (20)	*Not Tested*		*Not Tested*		*Not Tested*		9	(−38, 89)	−12	(−56, 78)
***Inhalation***											
**Near open fire**	37 (69)	**284**††	(62, 817)	**211**†	(35, 615)	**222**†††	(86, 458)	3	(−33, 56)	19	(−29, 101)
**Plastic accelerant**	6 (11)	135	(−37, 776)	27	(64, 347)	46	(−39, 248)	11	(−38, 98)	44	(−30, 195)
**Burned plastic trash**	7 (13)	−6	(−75, 260)	32	(−59, 322)	27	(−47, 204)	62	(−8, 186)	9	(−47, 125)
**Burned trash in outdoor burn pile**	13 (24)	**231**†	(23, 794)	**184**†	(11, 627)	**107**†	(5, 310)	9	(−31, 73)	10	(−37, 95)
**Burned plastic in outdoor burn pile**	10 (19)	**332**††	(58, 1082)	**251**†	(27, 872)	**134**†	(14, 382)	−16	(−50, 39)	<1	(−47, 86)

1 Cosmetics include foundation, compact powder, blush, eyeliner, mascara, lipstick, eyeshadow, and nail polish.

2 Hair products include gel, straightening products, conditioner, and/or shampoo.

† Significant at alpha < 0.05.

†† Significant at alpha < 0.01.

††† Significant at alpha < 0.001.

**Table 3:** Descriptive statistics for creatinine-adjusted (μg/g) BPA, phthalate, and PAH metabolites. N: number of samples; GM: geometric mean; CI: confidence interval; P: percentile; BPA: bisphenol A; MEP: mono-ethylhexyl phthalate; MBP: mono-*n*-butyl phthalate; MiBP: mono-isobutyl phthalate; MBzP: monobenzyl phthalate; MEHP: mono-2-ethylhexyl phthalate; MEHHP: mono-2-ethyl-5-hydroxyhexyl phthalate; MEOHP: mono-2-ethyl-5-oxohexyl phthalate; MECPP: mono-2-ethyl-5-carboxypentyl phthalate; MCMHP: mono-2-carboxymethylhexyl phthalate; PAH: polycyclic aromatic hydrocarbon; 1NAP: 1-naphthol; 2NAP: 2-naphthol; 2/3FLU: 2- and 3-hydroxyfluorene; 2/3PHE: 2- and 3-hydroxyphenanthrene; 1PHE: 1-hydroxyphenanthrene; 4PHE: 4-hydroxyphenanthrene; 1PYR: 1-hydroxpyrene.

Biomarker	N	GM (95% CI)	Median (IQR)	P90	P95	Minimum	Maximum
**BPA**	55	0.43 (0.35–0.52)	0.45 (0.24–0.68)	1.15	1.34	0.16	3.18
*Phthalate Metabolites*							
**MEP**	55	13.6 (9.62–19.2)	11.9 (6.24–25.8)	62.4	126	0.84	458
**MBP**	55	18.6 (13.0–26.6)	18.4 (6.88–52.8)	109	127	1.43	260
**MiBP**	55	6.11 (4.34–8.60)	6.40 (2.56–11.3)	29.0	74.9	0.55	123
**MBzP**	55	0.42 (0.53–0.34)	0.34 (0.25–0.67)	1.23	1.57	0.14	4.15
**MEHP**	55	2.56 (1.97–3.31)	2.65 (1.53–4.66)	7.60	10.4	0.16	31.0
**MEHHP**	55	21.5 (16.8–27.5)	19.3 (11.7–43.8)	63.6	94.5	1.08	118
**MEOHP**	55	11.3 (8.86–14.4)	9.04 (6.05–21.8)	38.5	49.4	0.65	59.7
**MECPP**	55	27.7 (22.1–34.6)	22.6 (17.1–48.1)	86.8	118	2.60	135
**MCMHP**	55	6.04 (4.91–7.43)	5.30 (3.71–9.19)	19.5	22.2	0.71	36.2
*PAH Metabolites*							
**1NAP**	55	12.5 (8.63–18.1)	17.3 (4.64–31.0)	41.5	68.1	0.54	939
**2NAP**	55	14.4 (10.6–19.4)	18.2 (7.34–30.7)	48.2	52.6	0.74	125
**2/3FLU**	55	2.36 (1.83–3.06)	2.59 (1.13–5.18)	7.07	7.51	0.19	11.0
**2/3PHE**	55	1.33 (1.04–1.69)	1.33 (0.69–2.78)	4.07	4.24	0.13	7.01
**1PHE**	55	0.73 (0.58–0.92)	0.78 (0.40–1.57)	1.86	2.15	0.07	2.76
**4PHE**	55	0.20 (0.16–0.25)	0.21 (0.11–0.40)	0.50	0.58	0.03	0.77
**1PYR**	55	1.34 (1.04–1.73)	1.37 (0.73–2.96)	4.23	5.81	0.11	6.78

For BPA, concentrations were 66% higher (p = 0.02) in participants who reported using cosmetics during the 48-h sample window. No additional significant associations were found between BPA and other potential sources, although the relationship between burning plastic trash in an open fire was associated with elevated BPA concentrations with nominal statistical significance (62% difference; p = 0.07).

Phthalate metabolite concentrations were 63% higher in participants who had reported using hair products (shampoo, conditioner, hair spray, etc.) during the 48-h period. The nine individual phthalate metabolites were regressed with the “hair products” variable and MEOHP was specifically associated with hair product use (p = 0.05; data not shown). The association between MBzP and hair products was nominally significant (p = 0.08; data not shown).

Personal PM_2.5_ concentrations were 2.84 times higher (p = 0.004) in participants who reported having been around an open fire. Concentrations were 2.31 times higher (p = 0.02) in participants who had burned trash in a burn pile, and 3.32 times higher (p = 0.007) in participants who said the burn pile contained plastic trash compared to participants who had reported not receiving the same exposures. Results are similar and statistically significant (p < 0.05) for personal BC and PAH metabolites for the same sources of exposure (see Table 2).

## 4 Discussion

We found that adolescent girls participating in our study had significantly reduced exposures to PM_2.5_, BC, and PAHs in the intervention arm compared to the control arm. We also measured detectable levels of phthalate and BPA metabolites in urine samples, some of which were higher than our reference NHANES population, and some of which were related to burning trash and cosmetics use.

We observed substantial median reductions of PM_2.5_ (−86%) and BC (−80%) exposures in intervention compared to control participants. These findings are consistent with similar studies comparing exposures in women who are primary cooks using biomass stoves compared to LPG stoves (Grajeda et al., 2020; Johnson et al., 2021; Thornburg et al., 2022; Weinstein et al., 2020). The results are comparable to the overall HAPIN study (Johnson et al., 2021), where a 74% reduction in exposures in intervention compared to control participants in Jalapa was reported.

Intervention participants also had substantially reduced (−72%) median urinary PAH concentrations compared to control participants. These findings are even more drastic than the 37% reduction found by Weinstein and colleagues in pregnant women in rural Guatemala using LPG compared to wood (Weinstein et al., 2020). This may be explained by the abundant free fuel delivered to homes in the HAPIN trial, while other studies limited the provision of free LPG fuel leaving houses to rely on a combination of fuels for daily cooking. Chen et al. also found significant reductions (p < 0.05) in urinary PAH concentrations in rural Chinese participants using LPG compared to wood, although percent reductions were not given (Chen et al., 2017).

Other than inhalation exposure from combustion sources, exposure to PAHs can occur through diet, specifically from eating grilled or charred foods (Hamidi et al., 2022). Tortillas are a staple food in Guatemala, and >50% of control participants and exactly 50% of intervention participants reported having eaten charred or burnt tortillas in the 48 h prior to urine sample collection. In this study, however, we did not find associations between participants’ report of eating charred or burnt tortillas and PAH concentrations as we did for PAHs and the inhalation exposures. This is consistent with the findings of similar studies (Weinstein et al., 2017, 2020).

BPA exposures in our participants were mainly attributed to general cosmetics use (p = 0.02), though borderline significance (p = 0.07) was found in relation to burning plastic trash in an open fire. Studies measuring BPA exposures in LMICs, especially in the context of HAP, are rare in the literature. A recent study that is relevant to ours was conducted by Rodríguez-Báez et al. (2022) in Mexico, where they measured urinary concentrations of PAHs, phthalates, and BPA in 45 indigenous women ages 24–79 years. In this study, behaviors that may lend to exposures to the pollutants of interest were smoking (9%), burning garbage (98%), using firewood for cooking (100%), and using plastic containers for food storage (100%). Median (IQR) creatinine-adjusted urinary BPA concentrations were 0.6 (0.4–2.0) μg/g creatinine, which is slightly higher than our findings of 0.45 (0.24–0.68) μg/g creatinine. In both studies, the urine sample was collected after an overnight fasting period (i.e., first morning void).

The biological half-life of both phthalates and BPA are believed to be only about 5 h (Galloway et al., 2018; Genuis et al., 2012; Wittassek and Angerer, 2008), meaning that substantial variations in urinary levels are likely; moreover, levels that are measured will relate to only current or recent exposures (Krishnan et al., 2010). This introduces substantial challenges and warrants caution when comparing results to other studies, especially ones that vary in design and population. Rodríguez-Báez's study differs from ours in that it was an observational study without air monitoring data, and no measures of association were reported between urinary metabolite concentrations and the potential sources of exposures listed.

Rodríguez-Báez et al. (2022) also measured urinary concentrations for four of the same phthalate metabolites as our study – MEHP, MBP, MiBP, and MBzP – in addition to the BPA already discussed. With the exception of MiBP, which was approximately six times higher in our participants, the findings of Rodríguez-Báez and colleagues were orders of magnitude higher than ours, and authors also noted that they were “impressively higher” than previous reports. In addition to differences in metabolism of phthalates in humans, another potential explanation the authors present for the high concentrations of phthalates in their study is the high prevalence of recycled plastics in the country. Mexico has been recognized as a leading recycler of plastics in the Latin America region, and recycled plastics are an important source of the parent compound DEHP, as it has been shown to be higher in recycled plastics compared to first-use plastics (Rodríguez-Báez et al., 2022). Overall, both our study and the Rodríguez-Báez study underscore the importance of assessing exposures in these understudied and vulnerable populations.

Compared to our reference NHANES population, five of the nine urinary phthalate metabolites analyzed (MBP, MEHHP, MEOHP, MEHP, and MECPP) were statistically significantly higher in our participants. MEHHP, MEOHP, MEHP, and MECPP have the same parent compound (DEHP), which is the most common member of the chemical class of phthalates and is widely used in plastic products (Carli et al., 2022). Participants in our study had variable personal care product and cosmetic use, but they reported that their community had no waste sanitation services and they observed an accumulation of plastic bottles and snack wrappers. These results illustrate the prevalence of plastics and their breakdown products in the environment, especially in places like Jalapa where municipal sanitation services are largely not available. Furthermore, while the United States and many countries of the European Union have restrictions for the use of some phthalates in consumer products, such regulatory infrastructure is not in place in many LMICs (Wang et al., 2019).

To our knowledge, this pilot study is the first to conduct comprehensive personal exposure assessment to HAP and measure urinary metabolites of plastics in adolescent girls in a rural setting where both biomass cookstoves and garbage burning are prevalent. Although a small study, we were able to leverage the HAPIN trial's participating households, which demonstrated excellent adherence to the LPG stove in intervention households (Quinn et al., 2021). Thus, we were able to see substantial reductions in the adolescents in the intervention group in the air pollutant results for PM_2.5_, BC, and PAHs, although the urinary biomarkers results for BPA and phthalates were less clear.

Cosmetic (foundation, compact powder, blush, eyeliner, mascara, lipstick, eyeshadow, and nail polish) and personal care product use during the 48-h time period was highly variable among our participants. More than half of our participants (53%) reported that they never use cosmetics, and only one participant used some cosmetics on a daily basis. Despite this, we were still able to see a relationship between cosmetic use and BPA concentrations.

Lotion, hair products, and deodorant were the personal care products used by at least 50% of participants during the sampling timeframe. For hair products, we were able to relate exposure to total phthalates and specifically, to MEOHP, but we did not observe associations between any other metabolite and personal care products. The short half-lives coupled with variable cosmetic and personal care product use, and small sample size of the study, may explain these findings.

Though we observed substantial reductions in the HAP data by study arm (PM_2.5_, BC, and PAHs), 36% of all participants (n = 20) reported burning trash including plastic at a frequency of at least twice per week during the baseline survey questionnaire. Whether a participant burned trash during our particular sample period was due to chance. Additionally, for those that reported burning trash within our 48-h sample period, they reported spending only about 10 min on average around the burn pile. For these reasons, the potential impacts of plastic trash burning in the community of Jalapa may not have been adequately characterized due to the inability to capture all burning events during the specified sample period and in this small sample size.

This study was an informative pilot study and, to our knowledge, one of the most comprehensive in terms of pollutants measured, but we were met with several limitations. In addition to the inherent limitations of short biological half-lives of some of the metabolites as previously discussed, this was a small, convenience sample, which may have led to either an over- or underestimation of the results. Moreover, cross-sectional studies such as ours only capture current exposures during a specific window of time and may not be representative of daily, cumulative exposures. The statistical analyses also relied heavily on survey data, which is inherently prone to recall and/or social desirability bias.

Given that BPA can leach into food and drinks, especially when the food and drinks are hot and come into contact with BPA-containing plastics, a missed opportunity we found with our questionnaire was not asking participants whether they had consumed hot beverages such as coffee from plastic cups. Furthermore, although we asked about frequency of garbage burning in participants’ households as part of the baseline survey questionnaire, garbage burning at any frequency was not part of the inclusion criteria, meaning that we only captured burning events in the households that happened to take place during the sample period. Despite this, we still were able to capture instances of garbage burning in our small study population and during the specified sample period. These specific limitations have been remedied in the subsequent village level cluster-randomized trial which expands upon this work in the same region of Guatemala (ECOLECTIVOS; NCT05130632).

Despite these limitations, this study showed that switching from biomass fuels to cleaner fuels such as LPG can significantly reduce exposures to harmful pollutants in young women of reproductive age. Cosmetics and some personal care products contributed to BPA and phthalate exposures, highlighting the ubiquitous nature of these chemicals in everyday products used by young women. More research is needed to accurately characterize cumulative exposures to these products in LMICs. Time-activity studies of HAP and household garbage burning, especially in these understudied areas, would also help elucidate the acute as well as cumulative exposures. Although it was beyond the scope of this study, it will be important for future studies to isolate the health impacts adolescent girls experience when exposed to air pollutants from garbage burning, including plastic.

## 5 Conclusion

This study characterized exposures to HAP from cookstoves and garbage burning, including plastic burning, among adolescent girls in rural Guatemala. We conducted 24-h personal air monitoring of PM_2.5_ and BC and analyzed urinary biomarkers of PAHs, phthalates, and BPA. We observed exposure reductions to PAHs, PM_2.5_, and BC in adolescents residing in homes that had been randomized to an LPG intervention compared to homes who continued cooking with traditional biomass stoves. We observed elevated levels of some urinary phthalate metabolites compared to U.S. participants in NHANES, and found detectable levels of BPA, which was associated with cosmetics use. This pilot study contributes to an area of the literature that is currently lacking, and paves the way for future work, namely the ECOLECTIVOS trial, in low resource settings where biomass and household garbage burning are prevalent.

## Funding

The HAPIN trial is funded by the U.S. National Institutes of Health (cooperative agreement 1UM1HL134590) in collaboration with the 10.13039/100021914Bill and Melinda Gates Foundation [OPP1131279]. Research reported in this publication was supported by the National Institute of Environmental Health Sciences of the National Institutes of Health under award number R01ES032009 and the EGHI Faculty Seed Grant funding provided by the Emory Global Health Institute at 10.13039/100006939Emory University. Additional support was provided by 10.13039/100000002NIH P30ES019776. The content is solely the responsibility of the authors and does not necessarily represent the official views of the U.S. National Institutes of Health or Emory Global Health Institute.

## CRediT authorship contribution statement

**Katherine A. Kearns:** Formal analysis, Investigation, Writing – original draft, Visualization. **Luke P. Naeher:** Writing – review & editing, Supervision. **John P. McCracken:** Conceptualization, Investigation, Writing – review & editing, Supervision, Funding acquisition, Project administration. **Dana Boyd Barr:** Investigation, Resources, Writing – review & editing. **Eri Saikawa:** Conceptualization, Investigation, Writing – review & editing, Supervision, Funding acquisition. **Mayari Hengstermann:** Conceptualization, Investigation, Writing – review & editing, Supervision. **Erick Mollinedo:** Investigation, Writing – review & editing. **Parinya Panuwet:** Investigation, Resources, Writing – review & editing. **Volha Yakimavets:** Investigation, Resources, Writing – review & editing. **Grace E. Lee:** Investigation, Resources, Writing – review & editing. **Lisa M. Thompson:** Conceptualization, Investigation, Writing – review & editing, Supervision, Funding acquisition, Project administration.

## Declaration of competing interest

The authors declare that they have no known competing financial interests or personal relationships that could have appeared to influence the work reported in this paper.

## Data Availability

Data will be made available on request.
